# AI-ADC: Channel and Spatial Attention-Based Contrastive Learning to Generate ADC Maps from T2W MRI for Prostate Cancer Detection

**DOI:** 10.3390/jpm14101047

**Published:** 2024-10-09

**Authors:** Kutsev Bengisu Ozyoruk, Stephanie A. Harmon, Nathan S. Lay, Enis C. Yilmaz, Ulas Bagci, Deborah E. Citrin, Bradford J. Wood, Peter A. Pinto, Peter L. Choyke, Baris Turkbey

**Affiliations:** 1 Artificial Intelligence Resource, Molecular Imaging Branch, National Cancer Institute, National Institutes of Health, Bethesda, MD 20892, USA; kutsev.ozyoruk@nih.gov (K.B.O.); stephanie.harmon@nih.gov (S.A.H.); nathan.lay@nih.gov (N.S.L.); eyilmaz@dmc.org (E.C.Y.); pchoyke@mail.nih.gov (P.L.C.); 2 Radiology and Biomedical Engineering Department, Northwestern University Feinberg School of Medicine, Chicago, IL 60611, USA; ulas.bagci@northwestern.edu; 3 Radiation Oncology Branch, National Cancer Institute, National Institutes of Health, Bethesda, MD 20814, USA; citrind@mail.nih.gov; 4 Center for Interventional Oncology, National Cancer Institute, National Institutes of Health, Bethesda, MD 20814, USA; bwood@cc.nih.gov; 5 Department of Radiology, Clinical Center, National Institutes of Health, Bethesda, MD 20814, USA; 6 Urologic Oncology Branch, National Cancer Institute, National Institutes of Health, Bethesda, MD 20814, USA; pintop@mail.nih.gov

**Keywords:** deep learning, prostate cancer, DWI, T2W MRI, generative artificial intelligence

## Abstract

Background/Objectives: Apparent Diffusion Coefficient (ADC) maps in prostate MRI can reveal tumor characteristics, but their accuracy can be compromised by artifacts related with patient motion or rectal gas associated distortions. To address these challenges, we propose a novel approach that utilizes a Generative Adversarial Network to synthesize ADC maps from T2-weighted magnetic resonance images (T2W MRI). Methods: By leveraging contrastive learning, our model accurately maps axial T2W MRI to ADC maps within the cropped region of the prostate organ boundary, capturing subtle variations and intricate structural details by learning similar and dissimilar pairs from two imaging modalities. We trained our model on a comprehensive dataset of unpaired T2-weighted images and ADC maps from 506 patients. In evaluating our model, named AI-ADC, we compared it against three state-of-the-art methods: CycleGAN, CUT, and StyTr2. Results: Our model demonstrated a higher mean Structural Similarity Index (SSIM) of 0.863 on a test dataset of 3240 2D MRI slices from 195 patients, compared to values of 0.855, 0.797, and 0.824 for CycleGAN, CUT, and StyTr2, respectively. Similarly, our model achieved a significantly lower Fréchet Inception Distance (FID) value of 31.992, compared to values of 43.458, 179.983, and 58.784 for the other three models, indicating its superior performance in generating ADC maps. Furthermore, we evaluated our model on 147 patients from the publicly available ProstateX dataset, where it demonstrated a higher SSIM of 0.647 and a lower FID of 113.876 compared to the other three models. Conclusions: These results highlight the efficacy of our proposed model in generating ADC maps from T2W MRI, showcasing its potential for enhancing clinical diagnostics and radiological workflows.

## 1. Introduction

Diffusion weighted imaging (DWI) was used primarily in the investigation of neurological disorders on magnetic resonance imaging (MRI), particularly in the clinical management of patients with acute cerebral ischemia [1]. However, following improvements in instrumentation, DWI also became a standard technique for body MRI, especially in the field of oncology [2]. What sets DWI apart is its ability to measure indices of water diffusion at the micrometer scale surpassing the usual millimetric spatial resolution of MR imaging [3]. For prostate cancer (PCa) imaging, two components of DWI data are used: high *b* value DWI and apparent diffusion coefficient (ADC) maps, which are calculated from DWI obtained at incrementally increasing *b* values. ADC maps are valuable not only in lesion detection but also in predicting PCa aggressiveness and in monitoring treatment response, particularly after therapies such as radiation therapy, chemotherapy, or androgen deprivation therapy [4,5,6,7]. Changes in ADC values over time can indicate treatment effectiveness and guide future treatment planning. ADC values are valuable in assessing the aggressiveness of PCa, which is crucial not only for staging but also for predicting patient outcome [8]. Despite advancements in MRI technology, the acquisition of high-fidelity ADC maps remains beset by challenges. Patient motion, even slight involuntary movements and rectal gas-related susceptibility, can easily introduce artifacts into DWI scans [9], which subsequently may result in image misalignment, thereby compromising the quality of ADC maps and their use in diagnostic workup. To address these challenges, deep learning-based image processing methods have recently received increased attention. Hu et al. [10] attempted to synthesize high *b*-value DWI (b = 1500 s/mm^2^), from the standard *b*-value DWI (*b* = 800 s/mm^2^ and *b* = 1000 s/mm^2^) via the CycleGAN approach. In another study, Hu et al. [11] synthesized ADC maps from diffusion-weighted images by deep learning (DL) approaches to avoid hardware dependencies, but to the best of our knowledge, there have been no prior attempts to produce ADC maps directly from T2W MRI.

DL-based solutions have shown promise in medical image synthesis [12]. Unsupervised image generation techniques like Pix2Pix [13] rely heavily on abundant high-quality data, often overlooking the intricate structure of image content, including textural details. Efforts have been made to enhance the content awareness of unsupervised GAN networks [14], but these attempts face challenges due to the substantial computational costs associated with improving edge awareness by demanding additional computation for both the generator and discriminator components. Recently, contrastive learning has emerged as a solution to address various challenges in unsupervised image transformation problems, aiming to create more robust latent space representations for the improved preservation of image content [15,16].

Currently, multiparametric MRI involving T2W MRI, DWI (including high “*b*” value diffusion and ADC maps), and dynamic contrast-enhanced MRI are obtained for PCa diagnostic workups, and this multiparametric approach requires approximately 30–40 min of scanner time, making it difficult and expensive to use MRI in a high-throughput mode. In this research, we propose a novel generative AI approach to tackle these challenges, enabling the acquisition of T2W MRI alone to generate ADC maps, potentially obviating the necessity for the actual acquisition of DWI and generation of ADC maps. 

The proposed GAN-based [17] AI-ADC method has the following novel contributions:

- Unpaired image-to-image translation: to generate ADC maps from T2W MRI without requiring explicit image pairing and annotation.
- A hybrid attention module: to drive the network attention to tumor-specific regions in the prostate by optimizing the future space.
- A self-regularization loss: to ensure that prostate boundary and other anatomical regions are reflected accurately in the synthesized ADC maps.

## 2. Materials and Methods

### 2.1. Prostate MRI Dataset

MRIs were obtained from a cohort of men undergoing mpMRI for suspicion of or known PCa. This retrospective study was approved by the Institutional Review Board of NIH, and written informed consent was obtained from all patients (ClinicalTrials.gov identifier: NCT03354416). MRIs were obtained at 3T (Ingenia Elition X, Philips, Best, Netherlands) using phased array surface coil with T2W turbo-spin-echo MRI, multiple *b*-value echo-planar DWI (0–1500 s/mm^2^), and ADC maps. The image quality of T2W MRI and ADC maps was evaluated by an expert radiologist with 20 years of experience in body imaging. The scoring system consists of three levels and the scoring criteria closely matches that of the Prostate Imaging Quality (PI-QUAL) system [18], encompassing both technical elements and visual evaluation parameters. Inclusion criteria for this study required scans that were considered capable of providing diagnostic information. Quality degradation was caused by one of several factors: motion, rectal gas-associated distortion, aliasing, and noise that prevented the ability to distinguish anatomical details such as the prostatic capsule, prostatic zones, intraprostatic lesions, sphincter muscles, and rectum/recto-prostatic space.

**Figure 1:**
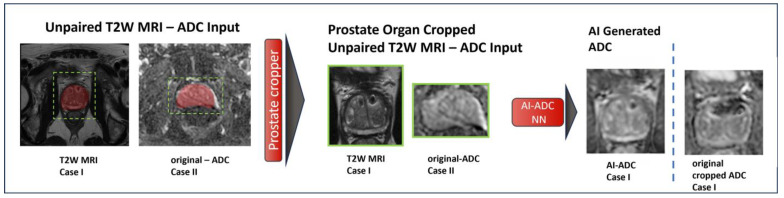
AI-ADC workflow. Upon completion of T2W MRI acquisition, either using in-house prostate segmentation AI model [19] or manual segmentation masks generated by radiologists, the prostate regions in T2W MRI and ADC maps are cropped. The cropped T2W MRI and ADC maps are fed as unpaired input images to the AI-ADC NN to produce high-fidelity prostate-focused ADC maps.

In the training dataset, we used 506 consecutive MRIs (n = 399 patients with acceptable image quality vs. n = 106 patients with high-quality image quality) in treatment-naïve patients. We tested the results on 195 randomly selected cases in the NIH cohort. We used axial T2W MRI, ADC maps, and manually derived prostate segmentation masks from each scan to train and evaluate models. The T2W MRI, ADC maps, and prostate segmentation masks were converted to NIfTI and resampled to have 0.5 mm × 0.5 mm × 3.0 mm voxel spacing. Images were normalized using z-score normalization. Based on the prostate segmentation masks that were prepared manually by the same study radiologist and via the prostate segmentation AI model developed in-house [19], we cropped both T2W MRI and ADC maps to include the prostate with minimal additional tissue, as overviewed in Figure 1.

We validated the results on ProstateX external validation data [20,21], which is publicly accessible and was employed in a global competition held from November 2016 to January 2017. It contains 347 examinations from 344 individuals, with 3 patients having undergone multiple scans. All imaging procedures were conducted at Radboud University Medical Center in Nijmegen, The Netherlands. Patients underwent T2-weighted, proton density-weighted, dynamic contrast-enhanced, and diffusion-weighted imaging using the 3 Tesla MAGNETOM Trio and Skyra scanner systems from Siemens Healthineers, Erlangen, Germany, without the utilization of an endorectal coil. T2W MRIs were acquired through a turbo spin echo sequence, featuring a 0.5 mm in-plane resolution and a 3.6 mm slice thickness. The DWI series were captured using a single-shot echo planar imaging sequence, offering a 2 mm in-plane resolution and a 3.6 mm slice thickness, incorporating diffusion-encoding gradients in three directions. The scanner software calculated ADC maps and *b* = 1400 DWI, with three *b*-values acquired (50, 400, and 800 s/mm^2^).

### 2.2. AI-ADC Method

In the proposed AI-ADC method, we employed a convolution block attention module integrated [22] ResNet-based [23] encoder with nine residual blocks as generator (G) to obtain a light-weight model considering its inference speed for real-time processing. We utilized PatchGAN [13] architecture as our discriminator. In Supplementary Materials Figure S1, one can find the detailed architecture. Least squares GAN loss [24] was used to ensure mode coverage and the avoidance of mode collapse. The adversarial loss function guided the learning process and diminished stylistic variations. This prompted the utilization of a noise contrastive estimation function, ensuring the preservation of content at the patch level. In this approach, we utilized a loss function based on patch-wise noise contrast to measure the similarity between a given T2W MRI and its corresponding ADC map. This involved selecting a patch from a generated image in the ADC domain and comparing it with a T2W MRI sample image patch located at the same position, aiming to create positive pairs if they exhibited resemblance. Conversely, patches that differed significantly were expected to form negative pairs. One positive with the corresponding input was mapped to L-dimensional real vector space, ℝ^L,^ $v,v^{+}\;\epsilon$ ℝ^L^. M negative non-corresponding input pairs were mapped to M × L dimensional real vector space, where $v^{-}$ ϵ ℝ^MxL^ and distance scaling by a temperature τ = 0.07.

The probability of positive examples selected over the negative ones was formulated as a cross-entropy loss and was calculated as:

(1) $$L\left(v,v^{+},v^{-}\right)=-\log\left[\frac{\exp\left(v\cdot v^{+}/\tau \right)}{\exp\left(v\cdot v^{+}/\tau \right)+\sum_{n=1}^{N}\;\exp\left(v\cdot v_{n}^{-}/\tau \right)}\right]$$

As per defined probability, $L\left(v,v^{+},v^{-}\right),$ it is anticipated that the patches extracted from the T2W MRI prostate region will exhibit a stronger association with the generated AI-ADC prostate region. We also benefited from Patch Noise Contrastive Estimation loss, $L_{\mathrm{PatchNCE}\;}$ [25], which was robust against noise in the data. To calculate this loss after L layers of interest are selected from the G, feature maps coming from the generator, G, are given as input to H, where H is projection head as defined in SimCLR [16] and follows Multilayer Perceptron (MLP) structure with one hidden layer. Similarly, the synthesized ADC images are encoded consecutively with these two networks G and H as features, z, {${\hat{z}}_{l}^{s}$} = {$H\;(G_{enc}^{l}$ (G(x)))}, where L is the layers of encoder indexed by l ∈ {1, …, L}. The patch-wise noise contrastive estimation loss, $L_{\mathrm{PatchNCE}\;}$, is defined based on the final features, {$z_{l}$}′s:

(2) $$L_{\mathrm{PatchNCE}\;}\left(G,H,X\right)=E_{x∼X}\sum_{l=1}^{L}\;\sum_{s=1}^{S_{l}}\;L\left({\hat{z}}_{l}^{s},z_{l}^{s},z_{l}^{S\backslash s}\right),$$

where s ∈ S = {1, …, $S_{l}$}, and $S_{l}$ is the number of spatial locations in each l layer. Thanks to this loss, we aimed to balance the fine-grained details with overall context. Additionally, to avoid the introduction of potentially misleading clinical information into the network, we imposed a penalty on deviations from original T2W MRIs using an L2 loss function at the pixel level, denoted as $L_{\mathrm{SR}}$ [26]:

(3) $$L_{\mathrm{SR}}\left(G,X\right)=∥X-G(X)∥.$$

In total, we defined our loss function, $L_{\text{AI-ADC}\;},$ as a summation of least squares GAN loss, $L_{GAN}$ [24], patchNCE, and self-regularization loss.

(4) $$L_{\text{AI-ADC}\;}\left(G,H,X\right)=+\left(G,X\right)+\lambda_{Y}L_{\mathrm{PatchNCE}\;}\left(G,H,Y\right)\;\;\;\;\;\;\;\;\;\;\;\;\;\;\;\;\;\;\;\;\;\;\;\;\;\;\;\;\;\;\;\;\;\;\;\;\;\;\;\;\;\;\;\;\;\;\;\;\;\;\;\;\;\;\;\;\;\;\;\;\;\;\;\;\;\;\;\;\;\;\;\;\;\;\;\;\;\;\;\;\;\;\;\;\;\;\;\;\;\;+\lambda_{X}L_{\mathrm{PatchNCE}\;}\left(G,H,X\right),$$

where λ_X_, and λ_Y_ are adjustable weight parameters for contrastive loss.

We used the mixture of two attention modules as a convolutional block attention module (CBAM): channel attention and spatial attention [22]. To achieve channel attention in the generator, we applied max pooling and average pooling operations to the feature maps that were fed MLP as an input. After elementwise summation operation, sigmoid activation function produced the output for channel attention block, $M_{C}\left(F_{i}\right)$:


(5) $$M_{C}\left(F_{i}\right)=\sigma \left(MLP\left(\left(AvgPool\left(F_{i}\right)\right)+MLP\left(\mathrm{MaxPool}\left(F_{i}\right)\right)\right)\right).$$

Similar to channel attention, we initially condensed the channel information of a feature map. Subsequently, we employed average and max pooling operations on the channel axis to produce features that were averaged $F_{\mathrm{Avg}\;}^{\prime S}$ and $F_{Max}^{\prime S}$ axes, respectively. Then, we applied the sigmoid function on top of convolution layer with 5 × 5 filter size, $C^{5\times 5}$,

(6) $$M_{S}\left(F^{\prime }\right)=\sigma \left(C^{5\times 5}\left(\left[F_{\mathrm{Avg}\;}^{\prime S},F_{Max}^{\prime S}\right]\right)\right)$$

The integration of channel and spatial attention module to ResNet block can be summarized as:

(7) $$F^{\prime \prime }=M_{S}\left(\left(M_{C}\left(F_{i}\right)⨂F_{i}\right)\right)⨂\;(M_{C}\left(F_{i}\right)⨂F_{i})$$

where ⨂ stands for elementwise multiplication.

We trained AI-ADC, CycleGAN [27], CUT [25], and StyTr^2^ [28] models for 5 epochs with batch size of 4 and experimented on in-house and ProstateX [20] datasets. In training, Adam optimizer [29] with a learning rate of 0.0002 and Glorot [30] weight initialization were employed.

### 2.3. Performance Metrics

The performance of the methods was measured with the Peak Signal-to-Noise Ratio (PSNR) [20], Structural Similarity Index (SSIM) [21], and Fréchet Inception Distance (FID) [22] metrics.

#### 2.3.1. Peak Signal-to-Noise Ratio

PSNR is defined as the ratio between the maximum possible power of a signal and the power of corrupting noise that affects the fidelity of its representation.

(8) $$\begin{array}{rr} PSNR & =10\times {log}_{10}\left(\frac{MAX_{I}^{2}}{MSE}\right)\\ & =20\cdot {log}_{10}\left(\frac{MAX_{I}}{\sqrt{MSE}}\right)\\ & =20\cdot {log}_{10}\left(MAX_{I}\right)-10\cdot {log}_{10}(MSE) \end{array}$$

(9) $$MSE=\frac{1}{MN}\sum_{i=1}^{M}\;\sum_{j=1}^{N}\;[I(i,j)-G(i,j){]}^{2}$$

where ${MAX}_{I}$ is the maximum pixel value of the original image, where I denotes the original image, and G stands for the AI-generated image.

#### 2.3.2. Structural Similarity Index

The Structural Similarity Index is a measure based on human perception for extracting structural information from a visual scene and consists of three parts: luminance (l), contrast (c), and structure (s). The SSIM is calculated on various windows of an image. The measure between two windows, x and y, of common size N × N is:


(10) $$SSIM(x,y)=\frac{\left(2\mu_{x}\mu_{y}+C_{1}\right)\left(2\sigma_{xy}+C_{2}\right)}{\left(\mu_{x}^{2}+\mu_{y}^{2}+C_{1}\right)\left(\sigma_{x}^{2}+\sigma_{y}^{2}+C_{2}\right)}$$

(11) $$l(x,y)=\frac{2\mu_{x}\mu_{y}+c_{1}}{\mu_{x}^{2}+\mu_{y}^{2}+c_{1}}$$

(12) $$c(x,y)=\frac{2\sigma_{x}\sigma_{y}+c_{2}}{\sigma_{x}^{2}+\sigma_{y}^{2}+c_{2}}$$

(13) $$s(x,y)=\frac{\sigma_{xy}+c_{3}}{\sigma_{x}\sigma_{y}+c_{3}}$$

where $\mu_{x}$ and $\mu_{y}$ are the average pixel values of windows x and y, respectively. $\sigma_{x}^{2}$ and $\sigma_{y}^{2}$ are the variances of windows x and y, respectively. $\sigma_{xy}$ is the covariance between x and y.

#### 2.3.3. Frechet Inception Distance

The FID calculates the distance between two Gaussian distributions: one representing the feature vectors of the real images with mean $\mu_{r}$ and covariance $\Sigma_{r}$ and the other representing the feature vectors of the generated images ($\mu_{g},\Sigma_{g}$).

(14) $$FID={\left\|\mu_{r}-\mu_{g}\right\|}^{2}+Tr\left(\Sigma_{r}+\Sigma_{g}-2{\left(\Sigma_{r}\Sigma_{g}\right)}^{0.5}\right)$$

where Tr denotes the trace of a matrix (the sum of all diagonal elements), and the square root of the covariance matrices is calculated using the eigenvalue decomposition.

#### 2.3.4. Dice Scores

Also known as Dice Similarity Coefficient (DSC), it is a statistic used to gauge the similarity between two sets of data. In medical image segmentation, it is used for the evaluation of segmentation annotations.

(15) $$\mathrm{Dice}=\frac{2\times |X\cap Y|}{\left|X|+|Y\right|}$$

where X and Y are the two sets being compared, typically representing the pixels or voxels in the ground truth segmentation and the predicted segmentation, respectively. |X| and |Y| are the total number of elements in each set. |X∩Y| is the size of the intersection of the two sets. 

## 3. Results

We benchmarked the performance of our proposed model against three state-of-the-art (SOTA) methods, CycleGAN [27], CUT [25], and StyTr^2^ [28], on 195 patients from our in-house dataset and 147 patients from the ProstateX dataset for external validation. Within the in-house test cohort, there were 38, 16, 58, 33, and 50 patients with PI-RADS scores of 1, 2, 3, 4, and 5, respectively. Within the ProstateX dataset, there were 32 PI-RADS 1 cases, 28 PI-RADS 2 cases, 30 PI-RADS 3 cases, 26 PI-RADS 4 cases, and 31 PI-RADS 5 cases.

We only included the scans in the training set if the radiologist clearly observed the prostate gland and the scan satisfied the quality requirements. The results are presented in terms of three metrics, PSNR, SSIM, and FID, for four methods CycleGAN, CUT, StyTr^2^, and AI-ADC. The results are presented in Table 1 for the in-house dataset for which we used expert annotations for cropping the prostate area from MRIs, in Table 2 for the same dataset but including in-house model-generated prostate boundary masks for prostate area cropping from MRIs, and in Table 3 for the ProstateX external validation dataset.

Even though the higher PSNR values usually indicate a better global image quality, this metric suffers from capturing the underlying structural details aligning with the human perspective. Therefore, we also evaluated the results in terms of SSIM, which ranged from −1 to 1 and provided a local measure of image quality. In addition to these two pixel-wise metrics, we compared the performance of models via FID scores, which also considered the distribution of high-level features. To compare each model’s performance, Wilcoxon signed rank test [23] was used. Although we observed a high variance for FID scores on the ProstateX and in-house datasets, the PSNR and SSIM values varied less compared to FID. When comparing the inference times per MRI slice for 152 by 152 and the performance metrics of various image-to-image translation models, AI-ADC emerged as highly suitable for real-time applications due to its optimal balance between high image quality (high PSNR and SSIM, and low FID) and low inference time (0.0065 ± 0.013 s). This contrasts with models like StyTr2 and CycleGAN, which, while offering quality transformations, do so at higher inference times (0.0197 ± 0.0034 and 0.0177 ± 0.0028 s, respectively), potentially limiting their use in real-time scenarios. Since the ProstateX dataset’s average cropped image size is 125 by 125, the inference time becomes shorter proportionally. The CycleGAN model first generator consists of 11.378 M and second generator also consists of the same amount of parameter. The AI-ADC model consists of 11.379 million parameters, and the CUT model consists of 11.378M. For StyTr^2^, we have 35.394 million parameters. 

We observed that the PSNR metric failed to adequately represent the structural variances in the generated images across all models in our in-house dataset. However, the PSNR values computed on the ProstateX dataset exhibited significant variations among them. Higher SSIM values, which are reported for AI-ADC, also refer to a greater similarity in terms of structure luminance, and contrast. Notably, the FID scores highlight the performance distinctions among the evaluated methods. 

**Figure 2:**
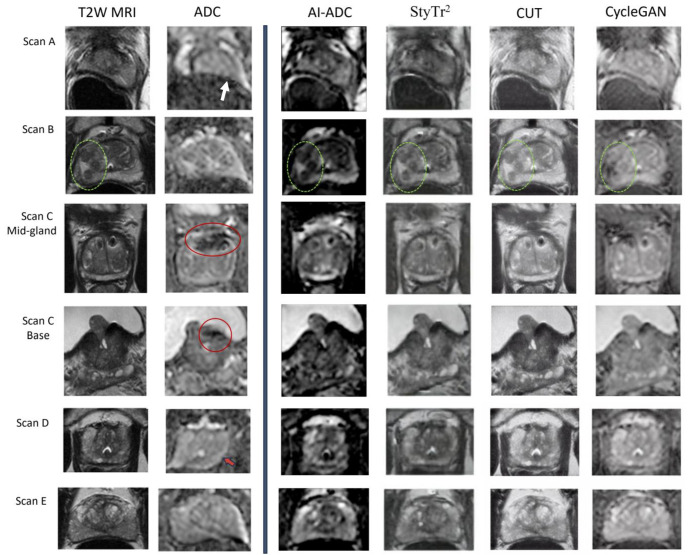
Qualitative MRI results across five different patients. The T2-weighted MRI (T2W MRI) and apparent diffusion coefficient (ADC) maps utilized in clinical evaluations are displayed on the left, and AI-generated ADC maps on the right are provided for comparison. Scan A: The presence of rectal gas significantly compromises the quality of the original ADC map, obscuring the posterior boundary of the prostate (white arrow). The AI-ADC map effectively delineates both the prostate boundary and intraprostatic zones; a comparative level of detail was not achieved by other AI-generated ADC maps. Scan B: The T2W MRI revealed three lesions in the right peripheral zone, which are not visible on the original ADC map. Except for the map produced by CycleGAN, all AI-generated ADC maps demonstrate these lesions (dashed green circles). Scan C: This scan shows a UroLift implant within the mid-to-base anterior transition zone of the prostate. Surrounding this implant, the original ADC map displays a significant artifact (red circle). In contrast, the AI-ADC map reveals detailed intra- and extraprostatic structures with minimal artifact presence. Scan D: In the original ADC map, the left posterior boundary of the prostate gland appears geometrically distorted (red arrow). The AI-generated ADC maps provide an enhanced delineation of prostate zones and outer boundaries. Scan E: The original ADC map shows a poorly defined and stretched left posterior prostate boundary. The AI-generated maps, particularly the AI-ADC, more accurately depict organ boundaries. Notably, the AI-ADC map more clearly highlights the hypointense capsule of a benign prostatic hyperplasia nodule in the left transition zone, compared to other generated images.

**Figure 3:**
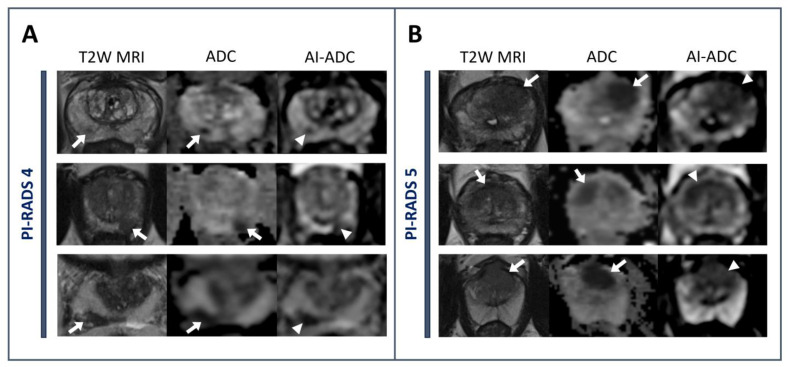
Representative cases from the ProstateX dataset with Prostate Imaging Reporting and Data System (PI-RADS) category 4 and category 5 lesions. PI-RADS category 4 lesions are located in right mid-peripheral zone, left apical peripheral zone, and right mid-base peripheral zone, from top to bottom, respectively (**A**). Lesions display a homogeneous hypointense signal on T2-weighted MRIs (T2W MRI) and original apparent diffusion coefficient (ADC) maps (arrows) and have a homogenous hypointense appearance on AI-ADC maps (arrowheads), whereas the lesions categorized as PI-RADS category 5 are situated in the left apical–mid anterior transition zone, midline apical–mid anterior transition zone, and midline apical–base anterior transition zone, from top to bottom, respectively (**B**). These lesions exhibit a hypointense signal on T2W MRI and the original ADC map (arrows) and also demonstrate a hypointense appearance on AI-ADC maps (arrowheads).

Based on SSIM and FID (*p* < 0.01), AI-ADC outperformed the cycle consistency based the CycleGAN approach both quantitatively and qualitatively, which performed the closest to AI-ADC. The qualitative results are exemplified using the NIH in-house dataset in Figure 2 and ProstateX dataset in Figure 3. In Figure 3, it is clearly demonstrated that the lesions exhibiting hypointense characteristics both in T2W MRI and ADC maps are generated as the hypointense area in AI-ADC maps. In Figure 2, we observed resilience against the existence of UroLift implant cases in Scan C, which is promising for the cases that almost never can obtain a clinically interpretable ADC map.

**Figure 4:**
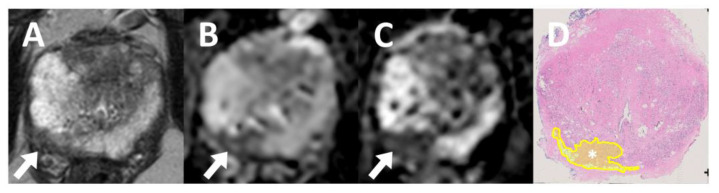
Histopathologic validation for AI-ADC maps. A 65-year-old male patient with a prostate-specific antigen level of 4.2 ng/mL presented with a Prostate Imaging Reporting and Data System (PI-RADS) category 5 lesion located in the right apical–mid peripheral zone. The lesion displays a hypointense signal on the in-house axial T2W MRI (**A**) and in the original ADC map (**B**) (arrows). In the AI-ADC map (**C**), the lesion displayed a markedly hypointense signal (arrow). In the whole-mount histopathology (**D**), the lesion (asterisk) was positive for International Society of Urological Pathology grade 3 prostate adenocarcinoma.

In Figure 4, we additionally verified the outcomes quantitatively using a radical prostatectomy-based histopathology sample. It was noted that the marked region for PI-RADS-5 lesions aligns with the hypointense area on the AI-ADC maps.

Apart from style and artifact issues in the ADC maps generated by the SOTA methods, hypointense cancer suspicious lesions were not well seen for the accurate diagnosis, so that the clinical value of the generated images remains suspicious. With the integration of channel attention, we aimed to optimize computational resources by reducing the processing load stemming from less informative features. Additionally, we benefit from spatial attention mechanisms to improve the sensitivity of the model in the abnormal regions. In the next step, by employing multi-reader studies we aim to prove the clinical adaptability of the AI-ADC method. Despite the success of our approach, the potential of this novel AI-ADC method relies on having prostate segmentation masks either generated by radiologists or automated organ segmentation models, which may not be always available. As a next step, we aim to enlarge the style transfer region to lose of this dependency. 

### In-House Prostate Organ Segmentor vs. Expert Annotation

**Figure 5:**
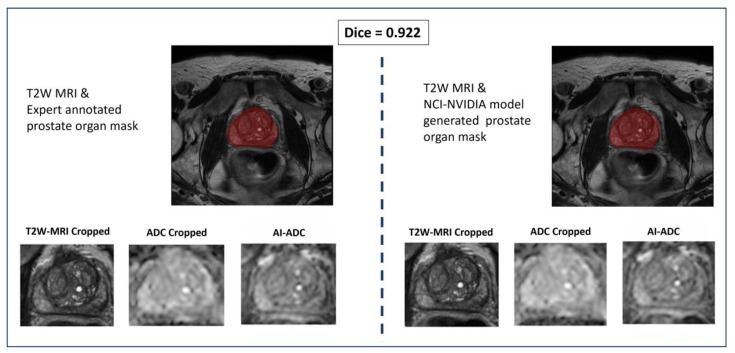
Expert annotation vs. AI segmentation. The left panel displays a T2W MRI with an expert-annotated prostate organ mask highlighted in red, and the right panel shows the same T2W MRI with a prostate organ mask generated by the in-house prostate segmentation model, also highlighted in red. Below each MRI, the corresponding cropped images show the T2W-MRI, ADC, and AI-generated ADC (AI-ADC) maps. The Dice coefficient of 0.922 indicates a high degree of similarity between the expert annotations and the AI-generated masks, demonstrating the effectiveness of the AI segmentation model in accurately delineating the prostate organ. Since the AI model and expert cropping area very close to each other, image synthesis was performed for a very similar area.

As detailed in the Methods Section, prostate segmentation is a needed step for our model to work and this can be achieved manually by a radiologist or by using AI models. We also evaluated our model’s performance when the prostate boundary is not manually delineated by radiologists, but instead generated by the in-house segmentation model [31]. Given the effectiveness of this model in prostate organ segmentation tasks, we report the Dice scores on the test dataset consisting of 195 patients, with a mean and standard deviation of 0.781 ± 0.286 for prostate segmentation. Figure 5 illustrates the segmentation mask generation for both the expert annotation pipeline and the AI segmentation pipeline, showcasing a test case where the Dice score is 0.922. In Table 2, we also showed that, if we used in-house segmentation model mask output to crop the prostate region, we can achieve similar PSNR, SSIM, and FID scores with the expert annotation usage.

## 4. Discussion

The key finding of our research is the development of a deep learning algorithm specifically designed to tackle the task of generating synthetic ADC maps from T2W MRI. Even though there are attempts in the literature to synthesize T2W MRI from ADC maps via U-Net, the results usually suffer from blurriness due to down-sampling and up-sampling operations [32]. With U-Net, the training requires a paired dataset where there is a direct correspondence between T2 and ADC maps. However, there are approaches in the literature that are not requiring paired input data, like CycleGAN. Even though GAN-based approaches like CycleGAN show a good performance in the task of generating a high b-value DWI from standard b-value DWI’s [10], or synthetic Computed Tomography from MRI to address the need for accurate dose calculation in MRI-only radiotherapy [33], in our application, the CycleGAN-produced ADC maps that suffered from undesired visual artifacts. For transformer-based StyTr^2^, a smaller image size may exacerbate its limitations, potentially resulting in more pronounced visual artifacts due to a reduced spatial resolution. Although transformer-based approaches like StyTr^2^ prove their performance in clinical applications [32], they may have more advantages in image classification and contextual understanding, which are the primary goals, as they excel in handling complex patterns and long-range dependencies. Apart from style and artifact issues in the ADC maps generated by the SOTA methods, hypointense lesions suspicious for cancer were not well visualized for accurate diagnosis, potentially limiting the clinical value of the generated images. One may wonder if it is possible to synthesize the images with other generative algorithms, such as diffusion probabilistic methods, where every step of the diffusion procedure is controlled better compared to GAN-based approaches, with fantastic results having been obtained with the Stable Diffusion algorithm [32]. While we are also enthusiastic about diffusion-based generative algorithms [34], at this stage, our overall goal is not fully overlapping with the heavy computational cost of diffusion strategies. Given the fact that we even simplified the CycleGAN procedure to make the inference in real time, diffusion approaches can be used in our framework once they are implemented in a less computationally burdened setting. Our methodology has quite a short inference time and, unlike the Cycle-GAN approach, our method omits the need for cycle consistency between the T2W MRI and ADC map domains, resulting in a reduction in the training time. With the integration of channel attention, we aimed to optimize computational resources by reducing the processing load stemming from less informative features. Additionally, in our approach with CBAM and ResNet-based encoder work, we benefit from spatial attention mechanisms to improve the sensitivity of the model in the abnormal regions.

**Figure 6:**
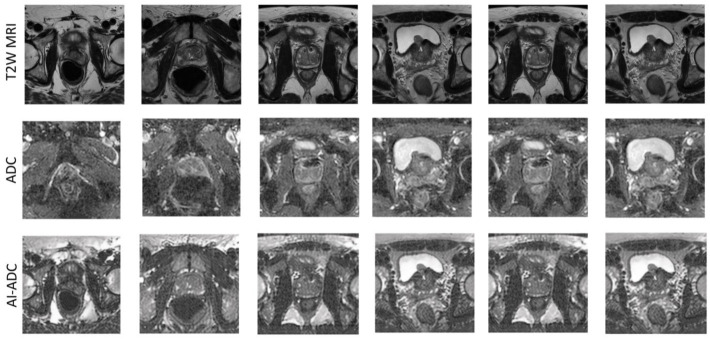
Failure analysis in unsegmented MRI approach. Initially, we utilized full-size T2W MRIs and ADC maps, resulting in failure due to variability in prostate size. Contrasting with our initial method, the current approach employs cropped images guided by the prostate segmentations.

In Figure 2, it is possible to quantitatively observe how spatial information in the T2W MRI is transferred throughout layers to keep structurally relevant details so that AI-ADC maps accurately depict the organ boundaries. In the next step, by employing multi-reader studies, we aim to prove the clinical adaptability of the AI-ADC method. Despite the success of our approach, the potential of this novel AI-ADC method relies on having prostate segmentation masks either generated by radiologists or automated organ segmentation models, which may not always be available. Producing prostate boundaries is a time-intensive task for radiologists and, with the additional experiment we run using our publicly available prostate segmentation AI model, we also showed that this step can be automatized. In Figure 6, we showed how the absence of the prostate boundary masks may result in failure even if we utilize the same dataset, same methodology, and same experimental setup. Including regions outside the prostate results in confusion in the style transfer by adding prostate-irrelevant structural details. At this stage, we are inspired by studies in synthesizing missing T1 MRI for brain tumor segmentation [34] since the details in brain MRIs can be comparable with those in prostate gland MRIs. 

The integration of advanced generative artificial intelligence models like AI-ADC for synthesizing ADC maps from T2-weighted MRI images marks a significant innovation in medical imaging, especially in the context of prostate cancer. AI-ADC’s superior performance over its counterparts, as demonstrated by the highest PSNR and SSIM values and the lowest FID score, underscores its ability to maintain image quality and structural integrity while closely mimicking the original image distribution. Although there appears to be a quantitative decrease in performance in the ProstateX dataset, the imaging technology used to gather this dataset, dating back to 2010, is relatively outdated. Therefore, this cannot be a direct indicator of the model’s generalizability. This model’s capabilities could revolutionize the diagnostic process by enhancing the accuracy of ADC maps, which are crucial for staging, treatment planning, and monitoring responses in prostate cancer care. Overall, our aim is to verify the effectiveness of the AI-ADC approach in a clinical environment. The clinical implementation of AI-ADC could potentially lead to a paradigm shift in how MRI is utilized in the early detection and continuous monitoring of prostate cancer. By providing high-quality ADC maps from T2-weighted images alone, this technology could reduce the need for multiple MRI modalities that currently compound patient discomfort and healthcare costs. This innovative approach not only mitigates the reliance on extensive imaging protocols but also promises a significant reduction in scanner time, making MRI more accessible and cost-effective as a routine screening tool. Our model has recently been adapted to generate ADC maps solely from T2-weighted (T2W) MR images, which can offer significant advantages for clinical applications. However, several studies have indicated that prostate-specific antigen (PSA) density is also a reliable indicator of the severity and biopsy outcomes of prostate cancer (PCa) [35], and AI can predict the biopsy outcome of PCa patients [36,37,38]. Exploring a multimodal approach that combines PSA density with T2W MRI data to produce ADC maps could be a promising direction for future research. This progress in AI-driven image synthesis represents a transformative step towards more precise and patient-centric imaging practices.

Our study has some limitations. First, this is a single-center study and the training dataset included MRIs from one hospital. However, we also tested our AI-model using an external dataset (ProstateX), which yielded promising results. Second, our experiment in this development study did not include AI-ADC with radiologist interaction. We are in process of running a multi-reader study to test the acceptance level of this unique approach by radiologists. Finally, we did not formally test the impact of AI-ADC on lesion detection, and this will be also further studied in our multi-reader study. 

## 5. Conclusions

In this work, we developed an automated and interpretable AI model for the generation of ADC maps from T2W MR images. This novel model may avoid the need for the acquisition of DWI and would be suitable for ADC map generation in practices and clinical studies.

## 6. Patents

An employee invention report was filed for this work in NCI, NIH.

## Figures and Tables

**Table 1:** SSIM, PSNR, and FID scores for our AI-ADC and SOTA methods on NIH Internal Dataset, which is cropped by prostate masks annotated by radiologists.

Models On Radiologists’ Masks	PSNR↑ *	SSIM↑	FID↓	Inference Time (s)
AI-ADC	**18.767 ± 3.357**	**0.870 ± 0.065**	**30.656**	0.0065 ± 0.013
StyTr^2^	18.289 ± 2.526	0.865 ± 0.076	61.286	0.0197 ± 0.0034
CUT	17.446 ± 2.315	0.801 ± 0.0082	174.752	**0.0048 ± 0.0005**
CycleGAN	17.950 ± 2.669	0.856 ± 0.047	58.071	0.177 ± 0.0028

* Upper arrows indicate that a higher score is better, and lower arrows signify that a lower score is better.

**Table 2:** SSIM, PSNR, and FID scores for our AI-ADC and SOTA methods on NIH Internal Dataset, which is cropped by the in-house prostate segmentation model. The Dice scores for the in-house model and radiologists’ annotations are calculated as 0.781 (±0.286). Although the Dice score is not so high, our model performs close to the radiologists’ annotated dataset.

Models On AI Masks	PSNR↑ *	SSIM↑	FID↓	Inference Time (s)
AI-ADC	**16.244 ± 2.413**	**0.863 ± 0.068**	**31.992**	0.0062 ± 0.0126
StyTr^2^	15.538 ± 2.398	0.824 ± 0.072	58.784	0.0196 ± 0.0033
CUT	14.500 ± 2.266	0.797 ± 0.079	179.983	**0.0048 ± 0.0004**
CycleGAN	16.082 ± 2.587	0.855 ± 0.071	43.458	0.0179 ± 0.0032

* Upper arrows indicate that a higher score is better, and lower arrows signify that a lower score is better.

**Table 3:** SSIM, PSNR, and FID scores for our AI-ADC and SOTA methods on the ProstateX External Validation Dataset.

Models	PSNR↑ *	SSIM↑	FID↓	Inference Time (s)
AI-ADC	**18.910 ± 1.287**	**0.647 ± 0.045**	**113.876**	0.0044 ± 0.013
StyTr^2^	16.049 ± 2.760	0.534 ± 0.143	120.393	0.0133 ± 0.0023
CUT	11.689 ± 2.040	0.467 ± 0.123	224.678	**0.0032 ± 0.0004**
CycleGAN	12.974 ± 2.593	0.512 ± 0.112	140.898	0.0120 ± 0.0019

* Upper arrows indicate that a higher score is better, and lower arrows signify that a lower score is better.

## Data Availability

The datasets generated during and/or analyzed during the current study are available from the corresponding author upon reasonable request.

## Supplementary Materials

The following supporting information can be downloaded at: https://www.mdpi.com/article/10.3390/jpm14101047/s1, Figure S1: AI-ADC Method Architecture.
