# Supplementary material for: AI-ADC: Channel and Spatial Attention-Based Contrastive Learning to Generate ADC Maps from T2W MRI for Prostate Cancer Detection
Source: J Pers Med. 2024 Oct 9;14(10):1047. doi: 10.3390/jpm14101047 (PMC11508265; doi:10.3390/jpm14101047)
Supplement: Supplementary file 1 [file jpm-14-01047-s001.zip › jpm-3208117-supplementary.pdf]

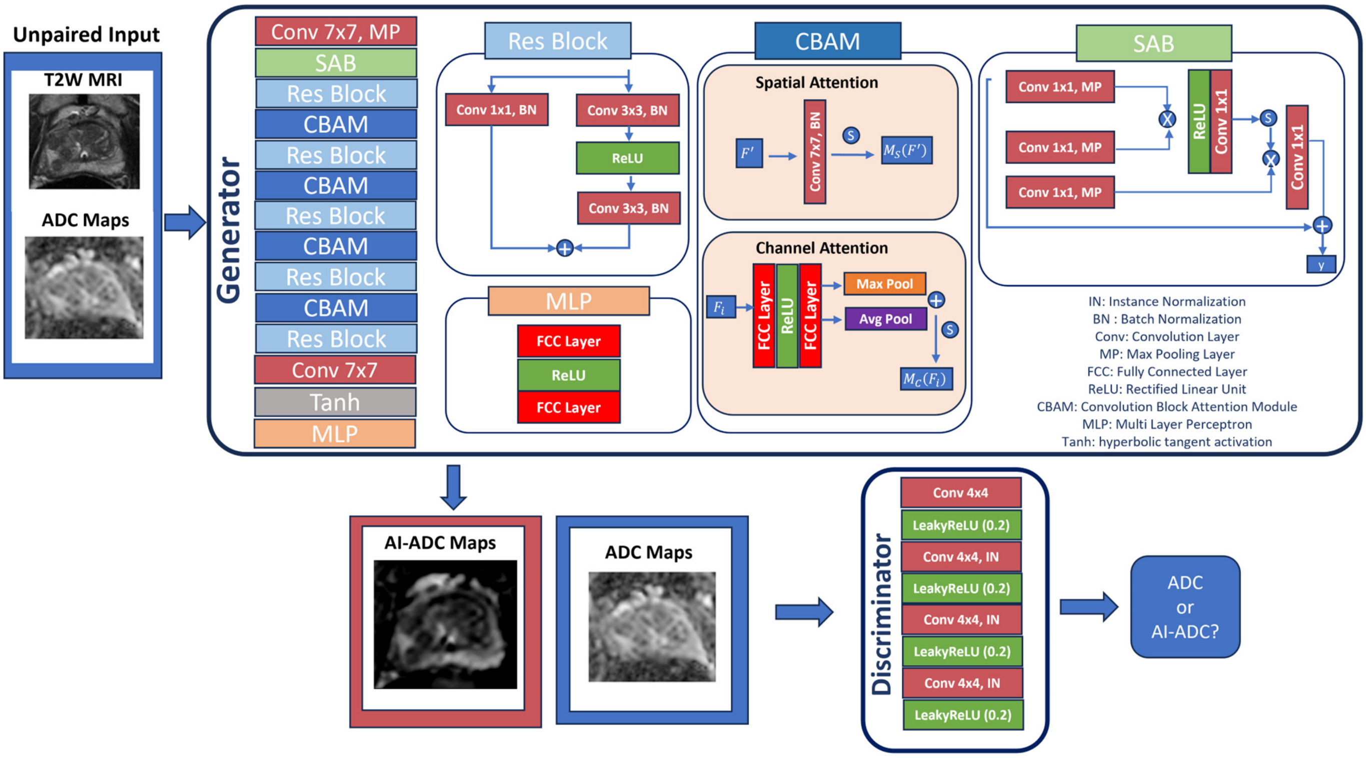

Supplementary Figure S1: AI-ADC Method Architecture. The unpaired T2W-MRI and ADC Maps are given as input to the AI-ADC generator, which features a ResNet-9 architecture embedded with SAB and CBAM. The generated AI-ADC maps and the original ADC maps are then fed into the discriminator, which attempts to determine which map is generated and which is the real ADC map. Thanks to the SAB module and the spatial attention module in CBAM, the spatial information of the lesion is enhanced, producing clinically relevant ADC maps.
